# Supplementary material for: ProteinShader: illustrative rendering of macromolecules
Source: BMC Struct Biol. 2009 Mar 30;9:19. doi: 10.1186/1472-6807-9-19 (PMC2672931; doi:10.1186/1472-6807-9-19)
Supplement: Additional file 1 — ProteinShader program without source code. This compressed file contains the complete ProteinShader program including associated libraries, but no source code. A README.txt file gives an overview of the ProteinShader distribution, and the index.html file in the help subdirectory has directions on getting started with the program as well as a set of tutorials. [file 1472-6807-9-19-S1.zip › ProteinShader-beta-0_9_4-binary/help/api/org/proteinshader/structure/class-use/Segment.html]

Uses of Class org.proteinshader.structure.Segment (ProteinShader API)


|  |  |  |
| --- | --- | --- |
| **Overview** \| **Package** \| **Class** \| **Use** \| **Tree** \| **Deprecated** \| **Index** \| **Help** | |  |
| PREV   NEXT | **FRAMES**    **NO FRAMES**     **All Classes** |  |


---


## **Uses of Class org.proteinshader.structure.Segment**

| Packages that use Segment | |
| --- | --- |
| **org.proteinshader.graphics** | Holds the drawing classes: Ribbon, Tube, FrenetFrames, Sphere, and Cylinder. |
| **org.proteinshader.graphics.adapter** | Holds the StructureToGraphics class, which is used to manage the use of the drawing classes and OpenGL display lists. |
| **org.proteinshader.graphics.displaylists** | Holds the classes needed to manage OpenGL display lists, which are used to cache reusable geometry for spheres, cylinders, ribbon segments, and tube segments. |
| **org.proteinshader.graphics.utils** | Holds a testing utility, class SegmentRenderer, which can be used for drawing ribbon and tube segments on-the-fly, rather than using cached geometry in the form of OpenGL display lists. |
| **org.proteinshader.structure** | Holds the classes that store information from a Protein Data Bank file: Structure, Model, Chain, AminoAcid, Heterogen, Water, Atom, Bond, Helix, BetaStrand, Loop, *etc*. |
| **org.proteinshader.structure.factory** | Holds the factory classes needed for generating the Segment objects that hold the information needed for rendering ribbon and tube segments. |
| **org.proteinshader.structure.visitor** | Holds Visitor classes that know how to traverse the hierarchy of objects held by class Structure. |
| **org.proteinshader.structure.visitor.modifiers** | Holds helper classes for the ModifierVisitor class, which is used to modify Drawable objects (Atoms, Bonds, and Segments). |

Uses of Segment in org.proteinshader.graphics

| Methods in org.proteinshader.graphics with parameters of type Segment | |
| --- | --- |
| `void` | `Silhouette.drawRibbon(GL gl, Segment s, SegmentListInfo info, boolean extraLines)`             Draws a translucent black silhoutte of a ribbon segment using jitter. |
| `void` | `Silhouette.drawTube(GL gl, Segment s, SegmentListInfo info, boolean extraLines)`             Draws a translucent black silhoutte of a tube segment using jitter. |

Uses of Segment in org.proteinshader.graphics.adapter

| Methods in org.proteinshader.graphics.adapter with parameters of type Segment | |
| --- | --- |
| `void` | `StructureToGraphics.draw(GL gl, Segment segment)`             Draws a Segment of a Region (GeneralLoop, Helix, or BetaStrand). |

Uses of Segment in org.proteinshader.graphics.displaylists

| Methods in org.proteinshader.graphics.displaylists with parameters of type Segment | |
| --- | --- |
| `SegmentListInfo` | `SegmentReferences.getSegmentListInfo(Segment segment)`             Returns the SegmentListInfo object that stores information on the OpenGL display lists that have been cached for the Segment given as an argument. |

Uses of Segment in org.proteinshader.graphics.utils

| Methods in org.proteinshader.graphics.utils with parameters of type Segment | |
| --- | --- |
| `void` | `SegmentRenderer.drawRibbon(GL gl, Segment s)`             Draws the broad surfaces of a ribbon segment and remembers info needed by any subsequent call to drawThinSidesOfRibbon(), drawStartCap(), or drawEndCap(). |
| `void` | `SegmentRenderer.drawTube(GL gl, Segment s)`             Draws the curved body of a tube segment and remembers info needed by any subsequent call to drawStartCap() or drawEndCap(). |

Uses of Segment in org.proteinshader.structure

| Methods in org.proteinshader.structure that return Segment | |
| --- | --- |
| `Segment` | `Segment.getNextSegment()`             Returns a reference to the next Segment with the same region so that the visiblity status of the next Segment can be checked before deciding to render a start cap for the current tube or ribbon segment. |
| `Segment[]` | `Model.getOpaqueSegments()`             Copies the list of opaque Segments into an array and returns the array. |
| `Segment` | `Segment.getPrevSegment()`             Returns a reference to the previous Segment with the same region so that the visiblity status of the previous Segment can be checked before deciding to render a start cap for the current tube or ribbon segment. |
| `Segment` | `Atom.getSegment()`             Returns the Segment the Atom is associated with (or null if the Atom is not associated with any Segment). |
| `Segment` | `Bond.getSegment()`             Returns the Segment the Bond is associated with (or null if the Bond is not associated with any Segment). |
| `Segment` | `Region.getSegment(String segmentID)`             Returns the Segment with the requested segmentID (if it exists). |
| `Segment` | `Model.getSegment(String segmentID, String regionID, RegionEnum regionType)`             Returns the requested Segment if it exists. |

| Methods in org.proteinshader.structure that return types with arguments of type Segment | |
| --- | --- |
| `Iterator<Segment>` | `Region.iteratorSegments()`             Returns an Iterator for the Segments owned by this Region. |

| Methods in org.proteinshader.structure with parameters of type Segment | |
| --- | --- |
| `void` | `Segment.setNextSegment(Segment next)`             Stores a reference to the next Segment within the same Region. |
| `void` | `Segment.setPrevSegment(Segment prev)`             Stores a reference to the previous Segment within the same Region. |
| `void` | `Atom.setSegment(Segment s)`             Sets the Segment the Atom is associated with. |
| `void` | `Bond.setSegment(Segment s)`             Sets the Segment the Bond is associated with. |

Uses of Segment in org.proteinshader.structure.factory

| Methods in org.proteinshader.structure.factory that return types with arguments of type Segment | |
| --- | --- |
| `LinkedHashMap<String,Segment>` | `SegmentFactory.createSegments(Region region)`             Creates the Segment objects for a Region. |
| `LinkedHashMap<String,Segment>` | `BetaStrandSegmentFactory.createSegments(Region region)`             After using the superclass createSegments() method, the LocalFrames held by some of the Segment objects will be modified so that beta-strands will have a pleated effect rather than a twisted ribbon appearance. |

Uses of Segment in org.proteinshader.structure.visitor

| Methods in org.proteinshader.structure.visitor with parameters of type Segment | |
| --- | --- |
| `void` | `ModifierVisitor.visit(Segment segment)`             If a SegmentModifier has been added, it will be used to modify the Segment. |
| `void` | `Visitor.visit(Segment segment)`             If debug is true, the segmentID will be printed to standard out. |
| `void` | `SFWriterVisitor.visit(Segment segment)`             Writes information on a Segment. |
| `void` | `VisibilityVisitor.visit(Segment segment)`             Tests the visiblity of the Segment in order to determine if it should be transferred to the opaque Segments or translucent Drawables list. |

| Method parameters in org.proteinshader.structure.visitor with type arguments of type Segment | |
| --- | --- |
| `void` | `VisibilityVisitor.setListsToFill(List<Segment> opaqueSegments, List<Drawable> opaqueAtomsAndBonds, List<Drawable> translucentList)`             Sets the lists to place opaque and translucent Drawable objects in. |

Uses of Segment in org.proteinshader.structure.visitor.modifiers

| Methods in org.proteinshader.structure.visitor.modifiers with parameters of type Segment | |
| --- | --- |
| `void` | `SegmentModifier.modify(Segment segment)`             Sets the patterns texture, halftoning texture, Segment color, or decoration type as requested by SegmentModifier methods, and then calls on modify() of the DrawableModifier superclass. |

---


|  |  |  |
| --- | --- | --- |
| **Overview** \| **Package** \| **Class** \| **Use** \| **Tree** \| **Deprecated** \| **Index** \| **Help** | |  |
| PREV   NEXT | **FRAMES**    **NO FRAMES**     **All Classes** |  |


---

# *Copyright © 2007-2008*
